# Context Matters: Multiple Novelty Tests Reveal Different Aspects of Shyness-Boldness in Farmed American Mink (*Neovison vison*)

**DOI:** 10.1371/journal.pone.0130474

**Published:** 2015-06-18

**Authors:** Christina Lehmkuhl Noer, Esther Kjær Needham, Ann-Sophie Wiese, Thorsten Johannes Skovbjerg Balsby, Torben Dabelsteen

**Affiliations:** 1 Behavioural Ecology Group, Section for Ecology & Evolution, Department of Biology, University of Copenhagen, Copenhagen, Denmark; 2 Research and Conservation, Copenhagen Zoo, Frederiksberg, Denmark; 3 Department of Bioscience, Marine Ecology, Aarhus University, Aarhus, Denmark; University of Pretoria, SOUTH AFRICA

## Abstract

Animal personality research is receiving increasing interest from related fields, such as evolutionary personality psychology. By merging the conceptual understanding of personality, the contributions to both fields of research may be enhanced. In this study, we investigate animal personality based on the definition of personality traits as underlying dispositional factors, which are not directly measurable, but which predispose individuals to react through different behavioural patterns. We investigated the shyness-boldness continuum reflected in the consistency of inter-individual variation in behavioural responses towards novelty in 47 farmed American mink (*Neovison vison*), which were raised in identical housing conditions. Different stages of approach behaviour towards novelty, and how these related within and across contexts, were explored. Our experimental design contained four tests: two novel object tests (non-social contexts) and two novel animated stimuli tests (social contexts). Our results showed consistency in shyness measures across multiple tests, indicating the existence of personality in farmed American mink. It was found that consistency in shyness measures differs across non-social and social contexts, as well as across the various stages in the approach towards novel objects, revealing that different aspects of shyness exist in the farmed American mink. To our knowledge this is the first study to reveal aspects of the shyness-boldness continuum in the American mink. Since the mink were raised in identical housing conditions, inherited factors may have been important in shaping the consistent inter-individual variation. Body weight and sex had no effect on the personality of the mink. Altogether, our results suggest that the shyness-boldness continuum cannot be explained by a simple underlying dispositional factor, but instead encompasses a broader term of hesitating behaviour that might comprise several different personality traits.

## Introduction

Animal personality is studied by measuring inter-individual variation in behavioural responses, which are consistent across time and contexts. Research in animal personality is receiving a lot of interest from related areas of research, such as evolutionary personality psychology research [1–3], since the evolved mechanisms that involve personality are likely to be similar across many different species, including humans [4]. Similar terminology is therefore used when studying individual differences in both human and animal behaviour, although the conceptual understanding of these terms seems to differ [5,6]. This difference relates in particular to the definition of ‘personality traits’. Behavioural ecologists often define a personality ‘trait’ using an empirical behavioural measure [5,6], whereas the psychological term ‘trait’ refers to a theoretical concept, which is not directly measurable but is inferred from the empirical measures [5].

As Coppens, Boer and Koolhaas [7] suggest, it might be helpful to merge the conceptual understanding of the terminology shared between the different fields in order for the research into animal personality to contribute to, as well as gain from, these other fields. This might also help to provide a framework for animal personality research, which could gain from a unification of the terminology and definitions for comparisons across studies [6,8]. An example of this is the shyness-boldness continuum [9–12], which is a widely studied aspect of animal personality. It is often described as an animal personality trait [13,14], although the evidence of its overall consistency across time and contexts is equivocal [10–12]. A range of different types of tests is used to study the shyness-boldness continuum, such as risky situations [8], novelty tests [9] and novel environments [15]. In addition, different behavioural measures are used to measure shy or bold behaviour, i.e. latencies in fish [16], the amount of novel objects explored in an arena in sheep [17] or activity levels when birds were confronted with a predator [18]. These many different approaches to the investigation of the shyness-boldness continuum may lead to different conclusions of the overall consistency of shyness-boldness. This was also shown by Carter et al. [12], who found that boldness according to one definition was not related to boldness using another definition. We agree with Carter et al. [12] on the importance of using multiple tests to measure aspects of animal personality and that the definition of shyness-boldness needs to be clear for comparisons across studies.

Furthermore we suggest to define animal personality traits as ‘underlying dispositional factors’, which are not directly measurable, but which cause individuals to react through different behavioural patterns [5]. According to this definition, the behavioural variables used to quantify the inter-individual differences in responses are not indeed the empirical measures of animal personality traits. Rather, these quantifiable variables are interpreted as reflecting measures of personality traits. Hence, animal personality traits, defined as dispositional factors, are the internal factors causing the consistency in behavioural responses, which means that environmental factors are not considered to have a direct impact on personality traits, except perhaps in the case of traumatization [19]. Different environmental stimuli may however induce the expression of different animal personality traits. This means that inter-individual variation in behavioural reactions can be expected to be consistent across those contexts, which induce the expression of the same personality traits.

This study investigates aspects of animal personality in the American mink (*Neovision vison)*. We aimed to measure shyness-boldness defined as the degree to which individuals hesitate when approaching novelty. We investigate how different stages, measured as latencies, in the approach towards novelty are related within and across contexts. Hence, we wish to use the term shyness-boldness as a simple description of the hesitating behaviour of individuals when approaching different types of novelty without initially labelling the tests.

Our study subjects are eight-month-old mink. As they are farmed and bred for fur quality, they are not selected for any particular behaviour, although some domestication has occurred, which may have reduced their overall fearfulness towards humans [20,21]. Since the mink have been raised in identical housing conditions, the inter-individual variation in behaviours is less likely to have been influenced by physical environmental factors. The American mink is a solitary carnivore, which makes this species suitable for studying individual reactions towards novelty without the confounding factor of separation anxiety. When investigating the personality of social animals, it is a major challenge to test individual’s reactions to novelty, because their reactions may be more influenced by the fact that they are isolated than by the stimulus presented to them [22].

The different contexts that we use to investigate the mink’s behaviour involve presenting the mink with different novel stimuli in their home cage. The tests are two novel object tests (one potentially less frightening and one potentially more frightening through the emission of sound) and two novel stimuli tests, which involve a conspecific (a mirror image and an unfamiliar live conspecific in an adjacent cage). Using this experimental design, we test the individuals in contexts that are similar in being novel, but with specific differences in content which are large enough to reduce the risk of the first trial substantially affecting the responses to the successive trials through habituation. Hence we investigate the consistency of the behavioural measures using the alternate form reliability approach [23], reducing the effects of habituation, which can be problematic when using the test-retest reliability method on novelty tests [24].

We aim to investigate the consistency of the shyness-boldness continuum using an experimental design that comprises four different contexts together with the measurement of different approach stages. In addition, by using farmed mink that are housed under identical conditions as our model animal we aim to investigate the inter-individual variation of this aspect of animal personality that is less influenced by variation in the environment and more influenced by inherited factors. Our results showed consistency in shyness measures across multiple tests, indicating the existence of personality in farmed American mink. It was found that consistency in shyness measures differs across non-social and social contexts, as well as across the various stages in the approach towards novel objects, revealing that different aspects of shyness exist in the farmed American mink.

## Methods

### Study animals and housing

The study animals were 47 captive wild-type American mink (*Neovison vison*) (12 females and 35 males) from Rørrendegård mink farm in Taastrup, Denmark. All were farm-reared, eight months old, and sexually inexperienced when tested in December 2012. The experimental setups were constructed in identical standard wire-mesh farm cages (W: 30 cm × H: 45 cm × L: 90 cm) connected to a wooden nest box with wire-mesh and straw on top (W: 28 cm × H: 20 cm × L: 23 cm) (manufactured by Hedensted Group, Denmark). Each cage was part of a series of six contiguous cages, joined at the longest side. Drinking water was provided *ad libitum* via a nipple drinker on a water pipe. All animals were fed a standard meat-based mink feed once daily, at around 4 p.m. The minimum temperature varied from -9 to +4°C.

### Behavioural tests

The following four novelty tests were carried out in the mentioned order.

#### Novel object test

A green cone-shaped dog toy weighing 129 g (H: 8.5 cm × D: 6.5 cm) was placed in the middle of the rear end of the home cage approximately 2 cm from the rear wire-mesh.

#### Novel object with sound test

A small radio-controlled car (11 cm long) was attached in a wire-mesh cage (H: 10.5 cm × W: 16.5 cm × L: 15.5 cm; weight 453 g) and placed approximately 2 cm from the rear end of the home cage. The first time the mink touched (or had its snout less than 1 cm away from) the wire-mesh cage containing the car, the observer activated the car from a distance. This made the car’s wheels turn, causing a sudden loud sound. The sound lasted for 30 seconds, after which the car was kept silent for the rest of the test.

#### Mirror test

A glass mirror (H: 30 cm × W: 28 cm) attached to a wooden plate (H: 40 cm × W: 28 cm × L: 1 cm) with height and width similar to the dimensions of the cage was suspended from the roof of the rear end of the cage. The mink had a full view of its own reflection as soon as it looked out of the nest box.

#### Conspecific test

A male mink in a small wire-mesh cage (H: 15 cm × W: 23 cm × L: 46 cm) was attached to the outside rear end of the home cage. Through the wire mesh, the test mink could see and make contact with this unfamiliar male mink, which could turn but only withdraw a few centimetres from the test mink’s cage. Each unfamiliar male was used only once and was never in the cage for longer than 15 minutes. Unfamiliar males were never included in the novelty tests.

Before each test (the pre-test procedure), the mink was kept inside its nest box for five minutes using a metal shutter to block the entrance hole while the novelty was placed in the back of the cage and a camera was mounted above the cage. Each test began by removing the metal shutter from the nest box, giving the mink access to its cage. The test lasted for eight minutes. During test time the neighbouring mink were kept in their nest boxes in order to not disturb the test animal. A dummy-test (test procedure without a novel stimulus) was performed on all mink before commencing the novelty tests. This was done in order to minimise the novelty effect of the pre-test procedure and to test if the individuals would respond to the removal of the shutter by looking out through the entrance hole of the nest box where they would be able to see the novelties. They all did so and were therefore deemed suitable for testing. To control the variable number of odours from the novelties, they were placed with three (the mirror) to four (the novel object and novel object with sound) different mink each morning before the test start. Each mink was exposed once to each test with at least two days between tests. All animals were subjected to all tests in the same order in order to avoid a reduction of the inter-individual variation in responsiveness due to habituation to the test situation and to the observers. The individuals were tested in a random order within each test using a systematic random design. The tests were performed at least two cages away from individuals being tested on the same day. All tests were performed outside the breeding season from 9 a.m. to 1 p.m. in December 2012.

### Ethics statement

According to the Danish law regarding animal experimentation, none of the novelty tests in this study required a license or special permission because they did not include any intrusive elements or measures that could potentially harm the animals (The Animal Experimentation Act no. 253, March 8^th^ 2013). The housing conditions at the commercial farm owned by the University of Copenhagen strictly follow the requirements of the Danish Animal Protection Act 2013, order no. 1734 of 2006 (Danish Ministry of Food, Agriculture and Fisheries). The University of Copenhagen gave permission to use the mink for this study. No animals were harmed during the non-invasive experiments and, after the experiments the animals were included in the normal farm production. The experimental farm is administered as a conventional mink farm according to Danish legislation (BEK 1428/15072002 & BEK 1734/22122006) administered by the Danish Veterinary and Food Administration. Animal experiments with mink have, when needed, been performed according to permit from the Danish Animal Experiment Inspectorate. Permit no. 2005/561-994.

### Observations and recording of behavioural variables

All tests were video-recorded using Logitech Portable Webcams C9 in order to minimize human contact. The eight-minute video recordings were analysed using continuous recording [25]. Latencies and other behavioural variables recorded as states and events were obtained from the video recordings. The latencies (in seconds) to approach, manipulate, or closely investigate the novelties were recorded during the four novel stimuli tests. However, only the latencies were included in further analyses because most other behaviours were performed by either too few individuals (less than two thirds) or were dependent on the latencies. All latencies were recorded from the test start until the behaviour was performed. The latencies were separated into independent variables afterwards by deducting the first latency from the second latency, and the first plus the second latencies from the third latency, etc. Table 1 shows an ethogram of the measured latencies defined test by test.

**Table 1:** Ethogram of behavioural variables recorded during the four novelty tests.

**Novel object test**	
Latencies to:	
***Out***	All four limbs outside the nest box.
***Snout***	Snout touching or less than 1 cm from the novel object for the first time. Measured from the time of *Out*.
***First manipulation***	First time biting or moving the novel object with snout or limb. Measured from the time of *Snout*.
**Novel object with sound test**	
Latencies to:	
***Out***	All four limbs outside the nest box.
***Snout 1***	Snout touching or less than 1 cm from the novel object for the first time. Measured from the time of *Out*.
***Snout 2***	Snout touching or less than 1 cm from the novel object for the first time after the sound. Measured from the time of *Snout 1*.
***First manipulation***	First time biting or moving the novel object with snout or limbs. Measured from the time of S*nout 2*.
**Mirror test**	
Latencies to:	
***Out***	All four limbs outside the nest box.
***Snout***	Snout touching or less than 1 cm from the mirror for the first time. Measured from the time of *Out*.
***First manipulation***	First time biting or moving the mirror with snout or limbs. Measured from the time of *Snout*.
**Conspecific test**	
Latencies to:	
***Out***	All four limbs outside the nest box.
***Snout***	Snout touching or less than 1 cm from the back end of the cage for the first time. Measured from the time of *Out*.
***First close investigation***	First time touching with limbs or biting the back end of the cage. Measured from the time of *Snout*.

### Statistical data analysis

#### Inter-observer reliability

Two observers watched all of the video recordings. Inter- and intra-observer reliability tests were performed before and after the actual observations on each behavioural variable. These were conducted using the Pearson product-moment correlation coefficient on parametric data and Spearman rank order coefficient on non-parametric data in order to test the linear relationship of the two observers for each type of latency. In addition, a t-test (parametric data) or a Mann-Whitney U test (non-parametric data) was used to determine if there were any significant differences between the two observers for each type of latency in order to check for any systematic differences in the observations. The criteria for passing the reliability tests were Pearson’s (or Spearman’s) r ≥ 0.7, p < 0.05, together with a t-test (or Mann-Whitney U test) with p > 0.05. Problematic variables found before the observations were redefined (the final definition is shown in the ethogram in Table 1) and rehearsed to avoid differences between observers. In addition, the two observers watched the same 23 and 24 mink in all four tests. The inter-observer reliability tests showed that all behavioural variables included in the analyses were reliable.

#### Missing not at random (MNAR) values

Of the total amount of 611 values (observations), 15 were characterised as MNAR due to truncation [18] by the fixed test duration. To avoid bias from deleting the (perhaps) most shy individuals from the dataset, and in order to use as much information as possible, estimated values for these 15 observations were used in the analyses. For six of the 15 latencies the minimum possible value was used. For example, if an individual did not come out of the nest box at all during the test period of 480 seconds, the latency to *Out* would be 480 seconds. Similarly, *if* an individual came out, but never got close to the stimulus during the test period, the latency to *Snout* would be 480 seconds minus the *value* of *Out*. In this way, we used as much kno*wn* information in the estimation of these missing values as possible. For the last nine unknown latencies an estimation was made using the FORECAST function in Microsoft Office Excel 2013 [26], which was based on the known latency (latencies) from the rest of the sample.

#### Analysis of data

We analysed the behavioural data in three steps. First, the behavioural data was reduced, test by test, into composite variables (principal components), grouping the latencies that were interrelated. Second, these principal components were interpreted, according to the grouping of latencies and the context, to represent behavioural responses. Third, the consistency of these responses was investigated by correlating the components across tests.

This approach can be described as a type of alternate form reliability method [23], which is an alternative method to the test-retest reliability method frequently used to determine consistency in animal personality research [5,27]. With the test-retest reliability method, the repeatability of single behavioural variables in repeated behavioural tests is calculated. This method proves to be problematic when using novelty tests. When presenting an animal with the same stimulus more than once the stimulus is already no longer novel at the second presentation [5,8,23]. The measured behaviour is likely to be more influenced by the rate of habituation or sensitisation of the individual rather than the reaction to novelty [8,22]. When using the alternate form reliability method the aim is to capture the personality trait reflected in the reaction towards novelty in two different tests, hence the stimulus used the second time must be sufficiently different that it is still novel. The different nature of the second stimulus may however motivate different types of behavioural variables, which all reflect the same personality trait. Alternatively, the same type of behavioural variable may reflect different personality traits depending on the stimuli, making the repeatability of single measurements dubious. Thus, the behavioural variables and behavioural responses should be evaluated within each test in order to determine which personality trait they may reflect if consistent across contexts. Therefore, the consistency of the behavioural responses should be validated by correlations across contexts.

#### Step 1: Reduction of variables

We reduced the variables into components using principal component analysis (PCA) [28] in SAS Enterprise Guide version 5. According to the assumptions of PCA, data is drawn from a multivariate normal distribution and variables should be continuous or measured on an interval scale [29]. All variables included in the PCA were normally distributed based on visual inspections of histograms and scatterplots, some after transformations (log10(x), log10(log10(x), square root, square root (N-x), Box-Cox transformation (lambda = 3)). The number of principal components (PCs) included in further analyses was based on the following criteria: their eigenvalue was larger than, or close to, one; the proportion explained by the PC should be equal to, or larger than, the proportion explained by a single variable (i.e. 0.25 for four variables); and visual inspections of the plateau in a scree plot. This resulted in one to two principal component scores (PC scores) per test.

#### Step 2: Interpretation of principal components

The PCs within each test were initially interpreted according to the loadings of the behavioural variables; the higher the loading of each behavioural variable, the stronger its effect on the PC.

#### Step 3: Consistency across tests

We tested for consistency of behavioural responses across contexts by correlating the PC scores across tests using Pearson’s product-moment correlation coefficient.

#### Effect of sex and body weight

In order to investigate whether sex and body weight could influence the inter-individual variation in the behavioural responses (reflected in the PCs), we performed a general linear model (GLM) as well as linear regressions using SAS. Since sex and body weight showed interdependence (female weight: 1580–2030 g, male weight: 2540–4420 g), the effect of body weight on the behavioural responses (i.e. the PC scores) was tested on males and females separately using linear regression. To test the effect of sex on the behavioural responses we used a GLM without interaction. Inspection of the residuals indicated no major deviations from normality. The regression and the GLM were performed using PROC GLM in SAS 9.3 (SAS Institute, Cary, NC).

## Results

### Principal component analyses test by test

Table 2 shows the results from the PCA for each of the four novelty tests. Most variance was explained by two PCs in both the novel object test and the novel object with sound test. All of these four PCs were initially interpreted as shyness. However, as the PCs within tests were uncorrelated due to the orthogonality of the PCs in a PCA, PCs on which the variables *Snout*, *Snout 1* and *Snout 2* had the highest loadings were labelled as Shyness 1 and PCs on which the variables *Out* and *First manipulation* had the highest loadings were labelled as Shyness 2 (Table 2). In both the mirror test and the conspecific test one PC explained most variance. Both of these PCs were also initially interpreted to reflect aspects of shyness and were both labelled Shyness (Table 2).

**Table 2:** Principal components and loadings of each behavioural variable from the four novelty tests.

**Novel object test**			**Novel object with sound test**		
**Principal components**	**1**	**2**	**Principal components**	**1**	**2**
*Eigenvalues*	*1*.*189*	*1*.*063*	*Eigenvalues*	*1*.*382*	*1*.*199*
*Proportion of variance*	*0*.*40*	*0*.*35*	*Proportion of variance*	*0*.*35*	*0*.*30*
***Out***	**-0.515**	**0.655**	***Out***	0.249	**0.660**
***Snout***	**0.755**	0.041	***Snout 1***	**0.663**	-.247
***First manipulation***	0.406	**0.755**	***Snout 2***	**0.607**	-.363
-	-	-	***First manipulation***	0.361	**0.610**
*Initial interpretation*	*Shyness 1*	*Shyness 2*		*Shyness 1*	*Shyness 2*
**Mirror test**			**Conspecific test**		
**Principal components**	**1**	-	**Principal components**	**1**	-
*Eigenvalues*	*1*.*926*	-	*Eigenvalues*	*1*.*623*	-
*Proportion of variance*	*0*.*64*	-	*Proportion of variance*	*0*.*54*	-
***Out***	**0.606**	-	***Out***	**0.619**	-
***Snout***	**0.643**	-	***Snout***	**0.576**	-
***First manipulation***	**0.469**	-	***First close investigation***	**0.533**	-
*Initial interpretation*	*Shyness*			*Shyness*	

The eigenvalue and the proportion of variance explained by each principal component are stated below each principal component. Our initial interpretations of the principal components are stated in the bottom row of each test. The loadings that are considered to be significant for the component have been marked in bold. Positive loadings indicate a higher degree of shyness and negative loadings a lower degree of shyness.

### Consistency of shyness

**Fig 1:**
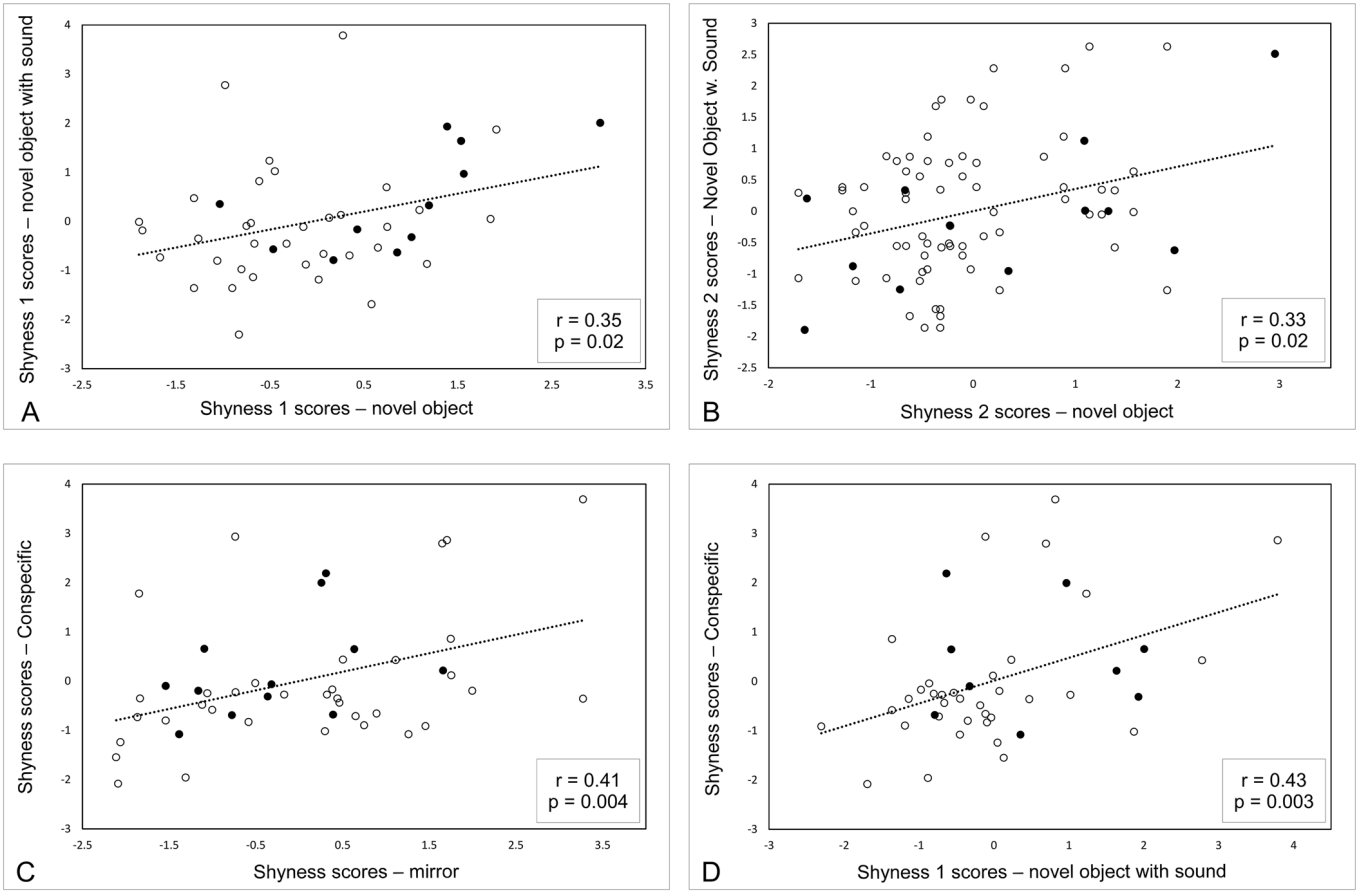
Scatterplots of the four significant correlations of shyness measures across tests. A) The Shyness 1 scores from the novel object with sound test vs the Shyness 1 scores from the novel object test. B) The Shyness 2 scores from the novel object with sound test vs the Shyness 2 scores from the novel object test. C) The Shyness scores from the conspecific test vs the Shyness scores from the mirror test. D) The Shyness scores from the conspecific test vs the Shyness 1 scores from the novel object with sound test. Filled-in circles represent females and open circles represent males. The dotted lines are linear trend lines (based on linear regression) added using Microsoft Office Excel 2013.

Each individual gained a PC score from each PC within tests, with a large PC score indicating a high degree of shyness. Thereafter the consistency of shyness was examined across tests by correlating these PC scores between tests. In the non-social contexts, represented by the two novel object tests, two out of four possible correlations were significant. Shyness 1 novel object with sound was positively correlated with Shyness 1 novel object (Pearson’s r47 = 0.35, p = 0.02) (Fig 1A), and Shyness 2 novel object with sound was positively correlated with Shyness 2 novel object (Pearson’s r47 = 0.33, p = 0.02) (Fig 1B). In the social contexts, represented by the two animated novelty tests, the one possible correlation was significant: Shyness conspecific was positively correlated with Shyness mirror (Pearson’s r47 = 0.41, p = 0.004) (Fig 1C). Across the social and non-social contexts, only one out of eight possible correlations was significant. Shyness conspecific was positively correlated with Shyness 1 novel object with sound (Pearson’s r47 = 0.43, p = 0.003) (Fig 1D). In total, four out of 13 correlations were significant which is more than expected by random (assuming α = 0.05) (see S1 Table for the results of all correlations).

### The influence of sex and body weight

The linear regressions of the PC scores representing shyness on body weight for males and females were all non-significant. Hence, body weight did not appear to influence the behavioural responses of either males or females (males: F_1, 30_ ≤1.48; p ≥ 0.23; females: F_1, 10_ ≤ 3.81; p ≥ 0.08) in any of the novelty tests.

The GLM overall model effect of sex was significant only on Shyness 1 in the novel object test (F_3, 42_ = 9.14; p = 0.004). The effect of sex was significant (F_1, 42_ = 9.14; p = 0.004) with females showing significantly higher PC scores than males (Lsmeans females: 0.75 ± 0.29, males: -0.26 ± 0.18). No other GLMs testing the effect of sex on the PC scores representing shyness were significant (F_3, 42_ ≤ 1. 07; p ≥ 0. 31).

## Discussion

Our results contribute to the field of animal personality research by showing that different latencies are consistent across multiple novelty tests, thus indicating the existence of personality in farmed American mink. Furthermore, our findings suggest that the consistency in shyness measures exist mainly within non-social and social contexts, respectively, as well as across different stages in the approach towards novel objects. We found that both the Shyness 1 and Shyness 2 measures in the novel object test were positively correlated with the Shyness 1 and 2 measures respectively in the novel object with sound test (Table 2). Furthermore, Shyness in the mirror test was positively correlated with Shyness in the conspecific test, and Shyness in the conspecific test was also positively correlated with Shyness 1 in the novel object with sound test. We speculate that the shyness measures may encompass different uncorrelated dispositional factors, which are consistent across contexts, although not expressed in all contexts. As the farmed mink have been raised in identical housing conditions, this indicates that the consistent inter-individual variation is more likely to be caused by inherited rather than physical environmental factors, although the possible effect of differences in litter size and composition as well as maternal care cannot be excluded.

This study contributes with a detailed investigation of animal behaviour characterised from shy to bold by first investigating different stages in the approach towards different types of novelty, then analysing these behaviours as potential personality measures within each test, and finally correlating across tests to prove consistency. Based on this approach, our results reveal that different aspects of shyness exist in the farmed American mink. In addition, our findings suggest that the shyness-boldness continuum is not one personality trait in itself, defined as an underlying dispositional factor, but rather a broader term of hesitating behaviours, which might encompass several different personality traits.

A study performed on wild baboons by Carter et al. [12] showed that boldness according to one definition was not related to boldness using another definition. The two boldness measures, anti-predator responses and latencies to approach novel food were uncorrelated and the authors suggest different interpretation of these measures. This supports the idea of context dependent behaviour, where the measure in itself does not describe a personality trait. We suggest that the attempt to describe personality traits relies on the interpretation of the underlying motivation of similar behaviour in different contexts.

As mentioned in the introduction there are challenges in comparing animal personality across species due to species specific behaviours such as solitary or social species. Comparing studies of the same species can also be difficult due to different approaches used when measuring, analysing as well as labelling different aspects of personality. Only a few other studies to date have investigated the personality in mink although using different approaches [20,30]. One study on European mink investigated approach behaviours towards a novel object as well as a mirror [30]. Haage et al. [30] found that fast approach to a novel object was related to fast approach to a mirror, which is in contrast to our findings. The explanation could be that the novelties were placed in their home environment, which is much larger than a farm cage. Hence, it is likely that the mirror from a distance was perceived as a novel object. When tested in a novel arena the European minks’ reaction towards the mirror did not correlate with those in the home enclosure. A study by Malmkvist & Hansen [20] investigated how two genetic lines of farmed American mink selected for their reaction to humans differed in their reaction to novel objects and unfamiliar mink. They found that the group of mink, which hesitated towards a novel object, also hesitated towards an unfamiliar mink. More studies on mink using a more unified approach and methodology are needed in the future.

In order to get a better understanding of the mechanisms of animal personality and the different aspects of shyness found in this study, we will in the following consider the underlying motivation of these shyness measures. The level of shyness in the mirror test, wherein the test mink potentially responds to a conspecific inside its own cage, may indicate a form of ‘social shyness’ or ‘social fearfulness’. Mirror tests have previously been suggested to induce ‘aggression’, ‘avoidance’ and ‘sociability’ in marmots [31]. We did not observe much overt aggressive behaviour from the individuals in this study. In the conspecific test, the behavioural responses of the test mink to the novel stimulus might reflect the level of sociability, defined as the degree to which the mink seeks the presence of a conspecific [8]. The threat intensity in the conspecific test is likely to be lower than in the mirror test since the test mink is separated from the conspecific by the familiar wire-mesh. Hence, the social contexts are characterised by social interactions or tolerance to a conspecific in a solitary species. This may add complexity to the underlying disposition of this behaviour, which is different in the non-social contexts.

Out of eight possible correlations between the social and non-social contexts, there was only one significant correlation between the Shyness scores from the conspecific test and the Shyness 1 scores in the novel object with sound test. As explained above the conspecific test is likely to induce a lower threat intensity than the mirror test. Thus, the Shyness scores from the conspecific test might be influenced by both the social behaviour directed at the conspecific in the rear end of the cage (which is a novel position for a neighbouring mink) and the level of insecurity in a novel situation similar to the novel object with sound test. In fact both the novel object with sound test and the conspecific test included a wire mesh cage separating the test animal from the stimuli. This prevented the test animal from direct contact with the novelty, inducing a factor of security, which was not present in the two other tests.

Interestingly, our results show different aspects of the shyness-boldness continuum within the novel object test and the novel object with sound test, as we find two uncorrelated measures of shyness within both of these tests. This could predict different functions of the latencies, where Shyness 1 is explained predominantly by the latency to *Snout*, whereas Shyness 2 is explained predominantly by the latencies to *Out* and *First manipulation* in both tests. The two aspects of shyness explain almost an equal amount of the variance and may represent different personality traits. To our knowledge, this is the first study to assess the different stages in the approach to novelty.

Latencies to approach a novel object have been interpreted as reflecting individual differences in general fearfulness [20,22,32]. Fearfulness can be described as the susceptibility of an individual to react to a variety of potentially threatening situations [32]. Koolhaas et al. [33,34] have demonstrated that animals seem to have different styles of coping when addressing stressors. It has been suggested that bold individuals are also more proactive while shy individuals have a reactive style of coping [35]. However, other studies indicate that shyness or fearfulness are not correlated with the style of coping [34,36,37]. We suggest that the two aspects of shyness (Shyness 1 and 2) found in the non-social tests could reflect coping style and fearfulness, respectively. Hence, individuals that can be described as fearful, due to perceiving the situation as potentially dangerous, may have different ways of coping. Faced with a potential threat, a fearful but proactive individual might exit the nest box quickly as it copes actively using the fight-or-flight response. It would spend time risk assessing outside the nest box leading to a longer approach time to the novel stimulus. This is in contrast to a fearful and reactive individual, which might cope passively using immobility, risk-assessing from inside the nest box leading to a longer latency to exit the nest box as well as to approach the novel stimulus [33]. Proactive individuals with lower levels of fear would be expected to have an active approach and exit the nest box quickly as well as approach the novel stimulus quickly. Reactive individuals with lower levels of fear have a more passive approach, leading to a slower exit of the nest box, however it will not hesitate much when approaching the novel stimulus. We speculate that this can reflect four different styles by which individuals respond (see Table 3).

**Table 3:** Interpretation of the separation of the latencies (*Out*, *First manipulation*, *Snout 1 and Snout 2*) on the two separate Shyness measures in the two novel object tests.

Style no.	Style description	*Out* and *First manipulation*	*Snout 1* and *Snout 2*
*1*	Less fearful, proactive	Short latencies	Short latencies
*2*	More fearful, proactive	Short latencies	Long latencies
*3*	Less fearful, reactive	Long latencies	Short latencies
*4*	More fearful, reactive	Long latencies	Long latencies

Styles 1–4 indicate an individual’s level of fearfulness and coping style for each of the four combinations of latencies.

The level of fear has a major role in all four styles (Table 3). However, the level of fear, as indicated by the different latencies, is not reflected in only short or long latencies because these may also reflect the style of coping. Taking this view, fearfulness and coping styles could be the underlying dispositional factors. This means that shyness reflects the hesitant behaviour observed, and that this behaviour is predominantly motivated by either the coping style or the level of fearfulness.

### The effect of sex and body weight

In order to investigate whether our findings can be explained by differences in physical parameters, linear regressions were performed to test whether the PC scores representing shyness were dependent on body weight for both males and females. The regressions were all non-significant proving that body weight did not appear to influence the behavioural responses of either males or females. Similar results have been found in European mink [30]. These findings indicate that the PC scores are indeed measuring aspects of the shyness personality trait that cannot simply be explained by smaller individuals reacting more shyly.

When testing for an effect of sex on all PC scores representing shyness, we only found an effect on Shyness 1 in the novel object test. The effect of sex was significant, with females showing significantly higher PC scores than males. This result indicates that females only react more shyly than males in the initial part of the novel object test, as they are to leave the nest box and touch the object for the first time. The explanation for this might be that the novel object test is the first test conducted out of the four and that the females might initially be shyer and more reluctant to novelty than males. The females might habituate fast as soon as they have had a closer look at the object i.e. touched it and realised there is no danger. In the study of personality in European mink [30] females generally scored lower than males in boldness measured as the minks’ reaction to novel objects and a mirror in the home enclosure. Sex differences were also found in the reaction to a mirror image in a novel arena in the breeding season only. However since there was no effect of sex on any other part of this test or the other tests there does not seem to be a general effect of sex on personality of mink in this study.

## Conclusions

Our results contribute to the field of animal personality by showing consistency across contexts in individual differences along the shyness-boldness continuum in the farmed American mink. By investigating shyness-boldness in approach behaviour across both non-social and social contexts, and with differentiated latencies, we also demonstrate the importance of clearly defining the shyness-boldness continuum when using different tests and behavioural measures. Thus, our study supports the concerns of Carter et al. [12] p. 607 when they ask: “How many studies that set out to assay one personality trait inadvertently assay another?”. In order to minimise these jingle-jangle fallacies [12], we suggest to define animal personality traits as underlying dispositional factors. In addition, this study highlights the limitations of using shyness-boldness continuum for describing animal personality traits. Instead, it can be used to describe the observed behaviour, keeping in mind that these behaviours may be motivated by different animal personality traits. In this study, we suggest that the shyness-boldness measured in the social contexts is motivated by sociability or ‘social shyness’. The shyness-boldness measured in the non-social contexts is suggested to be motivated by two uncorrelated personality traits; fearfulness and coping styles. These may be expressed through different behavioural styles of the individuals. It is likely that these dispositional factors are inherited, although our study did not account for the possible effect of the variation in maternal care. The effect of maternal care and the consistency of these different shyness measures across time and season would be interesting to investigate in future studies. By considering each context separately, we have shown differentiated consistent behaviour in the level of shyness in novel object tests and across non-social and social tests. Therefore, when investigating animal personality by measuring the inter-individual behavioural responses we need to consider carefully the context and the possible different ways by which the underlying dispositional factors of personality can be expressed.

## Supporting Information

S1 File Raw data of the latency in seconds of recorded behaviours from the four novelty tests used in PCA analyses as well as the physical parameters Sex and Body weight in gram for all mink. See the Ethogram in Table 1 for descriptions of the behavioural variables recorded.
(XLSX)

S1 Table Correlation matrix of the principal components interpreted as shyness. Values without brackets are Pearson's r-values. Values in brackets are the corresponding p-values. Significant values are shown in bold.
(DOCX)
