# Supplementary material for: Context Matters: Multiple Novelty Tests Reveal Different Aspects of Shyness-Boldness in Farmed American Mink (Neovison vison)
Source: PLoS One. 2015 Jun 18;10(6):e0130474. doi: 10.1371/journal.pone.0130474 (PMC4472346; doi:10.1371/journal.pone.0130474)
Supplement: S1 Table — Values without brackets are Pearson's r-values. Values in brackets are the corresponding p-values. Significant values are shown in bold. (DOCX) [file pone.0130474.s002.docx]

|  |  | **Novel object with sound** | | **Mirror** | **Conspecific** |
| --- | --- | --- | --- | --- | --- |
|  |  | **Shyness 1** | **Shyness 2** | **Shyness** | **Shyness** |
| **Novel object** | **Shyness 1** | **0.35**  **(p = 0.02)** | -0.20  (p = 0.19) | -0.14  (p = 0.34) | 0.11  (p = 0.46) |
|  | **Shyness 2** | 0.18  (p = 0.22) | **0.33**  **(p = 0.02)** | -0.16  (p = 0.28) | 0.13  (p = 0.39) |
| **Novel object with sound** | **Shyness 1** | - | - | 0.20  (p = 0.17) | **0.43**  **(p = 0.003)** |
|  | **Shyness 2** | - | - | 0.02  (p = 0.92) | 0.14  (p = 0.36) |
| **Mirror** | **Shyness** | - | - |  | **0.41**  **(p = 0.004)** |
